# Achievement of remission after first-episode psychosis in youth

**DOI:** 10.1192/j.eurpsy.2021.2125

**Published:** 2021-08-13

**Authors:** V. Kaleda, D. Tikhonov

**Affiliations:** 1 Department Of Youth Psychiatry, FSBSI «Mental Health Research Centre», Moscow, Russian Federation; 2 Department Of Youth Psychiatry, Mental Health Research Centre, Moscow, Russian Federation

**Keywords:** achievement of remission, youth psychiatry, First episode psychosis

## Abstract

**Introduction:**

Dynamic assessment of remission achievement after first-episode psychosis (FEP) is necessary for early detection of post-psychotic depression, negative symptoms and changes in personality traits. The latter allows to decrease suicide risk and optimize treatment and social rehabilitation.

**Objectives:**

We aimed to analyze achievement of remission after FEP in youth and to define prognostic criteria for psychosis outcome.

**Methods:**

Fifty-six patients (16-25 y.o., mean age 19,8 ±2,5 y.o.) after FEP have been receiving follow-up outpatient visits for 3 years. PANSS was applied to assess psychotic symptoms. Depressive symptoms were assessed with HAMD-21.

**Results:**

Remission achievement after FEP is a three-stage process. The stage of reduction and modification of psychotic symptoms is characterized by diminishing personality deterioration and decrease of leading positive symptoms. The second stage, stabilization, is defined through the presence of depressive symptoms with positive (HAMD-21 17,49 ± 7,49) and negative affectivity (HAMD-21 23,68 ± 9,24) with preponderance of emotional, volitional, and cognitive deficits as well as high suicide risk. The third stage, reintegration, is characterized by the combination of negative symptoms with preserved personality resources. There are three reintegration trajectories, with predominant affective or negative symptoms or personality deficits. Mean decrease of PANSS scores was 54,88 ± 6,17 during the overall remission. In the majority of cases (62,5%) the stage of reintegration was finished with the achievement of high-quality remission, coinciding with international remission criteria. The study was supported by RFBR grant 18-013-01214

**Conclusions:**

Our approach to remission assessment allowed us to decrease suicide risk and to provide optimal treatment.

**Disclosure:**

No significant relationships.

